# COVID-19 vaccine hesitancy worldwide and its associated factors: a systematic review and meta-analysis

**DOI:** 10.1016/j.soh.2023.100048

**Published:** 2023-11-14

**Authors:** Matin Baghani, Farzan Fathalizade, Amir Hossein Loghman, Noosha Samieefar, Farbod Ghobadinezhad, Ronak Rashedi, Hediyeh Baghsheikhi, Fatemeh Sodeifian, Milad Rahimzadegan, Meisam Akhlaghdoust

**Affiliations:** a Functional Neurosurgery Research Center, Shohada Tajrish Comprehensive Neurosurgical Center of Excellence, Shahid Beheshti University of Medical Sciences, Tehran, Iran; b Cognitive and Neuroscience Research Center (CNRC), Tehran Medical Sciences, Islamic Azad University, Tehran, Iran; c USERN Office, Shahid Beheshti University of Medical Sciences, Tehran, Iran; d Student Research Committee, School of Medicine, Shahid Beheshti University of Medical Sciences, Tehran, Iran; e Student Research Committee, School of Medicine, Kermanshah University of Medical Sciences, Kermanshah, Iran; f USERN Office, Kermanshah University of Medical Sciences, Kermanshah, Iran; g USERN Office, Functional Neurosurgery Research Center, Shahid Beheshti University of Medical Sciences, Tehran, Iran; h Network of Interdisciplinarity in Neonates and Infants (NINI), Universal Scientific Education and Research Network (USERN), Tehran, Iran

**Keywords:** Vaccine hesitancy, Vaccine acceptance, COVID-19, SARS-CoV-2, Coronavirus, Meta-analysis

## Abstract

**Introduction:**

The severe acute respiratory syndrome coronavirus 2 (SARS-CoV-2) pandemic has taken a toll on humans, and the development of effective vaccines has been a promising tool to end the pandemic. However, for a vaccination program to be successful, a considerable proportion of the community must be vaccinated. Hence, public acceptance of coronavirus disease 2019 (COVID-19) vaccines has become the key to controlling the pandemic. Recent studies have shown vaccine hesitancy increasing over time. This systematic review aims to evaluate the COVID-19 vaccine hesitancy rate and related factors in different communities.

**Method:**

A comprehensive search was performed in MEDLINE (via PubMed), Scopus, and Web of Science from January 1, 2019 to January 31, 2022. All relevant descriptive and observational studies (cross-sectional and longitudinal) on vaccine hesitancy and acceptance were included in this systematic review. In the meta-analysis, odds ratio (OR) was used to assess the effects of population characteristics on vaccine hesitancy, and event rate (acceptance rate) was the effect measure for overall acceptance. Publication bias was assessed using the funnel plot, Egger's test, and trim-and-fill methods.

**Result:**

A total of 135 out of 6,417 studies were included after screening. A meta-analysis of 114 studies, including 849,911 participants, showed an overall acceptance rate of 63.1%. In addition, men, married individuals, educated people, those with a history of flu vaccination, those with higher income levels, those with comorbidities, and people living in urban areas were less hesitant.

**Conclusion:**

Increasing public awareness of the importance of COVID-19 vaccines in overcoming the pandemic is crucial. Being men, living in an urban region, being married or educated, having a history of influenza vaccination, having a higher level of income status, and having a history of comorbidities are associated with higher COVID-19 vaccine acceptance.

## 1 Introduction

Severe acute respiratory syndrome coronavirus 2 (SARS-CoV-2) first emerged in 2020 Pandemic in December 2019 and soon became a global concern owing to its high transmissibility; it was announced as a pandemic by the World Health Organization (WHO) in March 2020. Various strategies have been considered to prevent further transmission. Obligatory wearing of face masks, social distancing, travel restrictions, lockdowns, and quarantine were the first steps taken by most countries worldwide. Despite being partially successful in limiting further disease transmission, these strategies resulted in tremendous economic devastation [1,2].

The constant emergence of new variants of SARS-CoV-2, which are either more transmissible or cause greater morbidity and mortality, further raised concerns about a more cost-effective solution. Therefore, ever since vaccines became available and approved for use at the end of 2020, they have been considered the most effective and crucial strategy to fight coronavirus disease 2019 (COVID-19) [[3], [4], [5]].

Nevertheless, for a vaccination program to be successful, a considerable proportion of the community must be vaccinated. At least 70% of each community must be fully vaccinated to achieve herd immunity, and this number may be greater based on the vaccine type and transmissibility of the circulating variants [4,6,7]. Hence, public acceptance of COVID-19 vaccines has become the key to controlling the pandemic. It is therefore important to ensure that maximum vaccine coverage is reached by making vaccines accessible and affordable and increasing public awareness to achieve maximum vaccine acceptance [8].

However, studies have shown that the rate of vaccine hesitancy has increased over time, making it the most important concern in the fight against COVID-19. The WHO declared vaccine hesitancy, defined as the refusal to get vaccinated despite the availability of vaccines, as one of the top 10 global health threats in 2019. This growing hesitancy may be because of an altered perception of the disease risk, uncertainty about available vaccines, fear of side effects, misinformation, and the spread of fake news [3,8,9].

Perception of health risk is strongly associated with vaccine hesitancy. Consequently, for public health to improve people's knowledge and attitudes toward vaccination, it is necessary to first understand this phenomenon's social, demographic, and psychological determinants. Furthermore, the language and communication strategies or media used to convey a health message influence how the vaccine is received. To accomplish this, all authorities involved in health communication must work together to produce clear and coherent messages [10].

With vaccines being the most important and effective weapon in the battle against COVID-19, it is essential to address the factors contributing to vaccine hesitancy and attempt to increase the rate of vaccine acceptance in the community.

Herein, a systematic review was performed to detect the intention to receive COVID-19 vaccines among different communities and identify different population characteristics and factors associated with COVID-19 vaccine hesitancy.

## 2 Method

### 2.1 Data source and search strategy

On February 15, 2022, MB searched MEDLINE (via PubMed), Scopus, and Web of Science to find relevant articles. The search was limited to records from January 1, 2019 to January 31, 2022, and no language restrictions or filters were applied to search the databases. The search strategy for PubMed was as follows: (((((((“Vaccination Hesitancy”) OR (“Vaccination Refusal”)) OR (“Vaccine Hesitancies”)) OR (“Acceptance of vaccination”)) OR (“Vaccination Delay”)) OR (Vaccine Hesitancy)) AND (((((((“SARS2 Vaccine”) OR (“COVID-19 Vaccines”)) OR (“SARS-CoV-2 Vaccine”)) OR (”2019-nCoV Vaccine”)) OR (”2019 Novel Coronavirus Vaccine”)) OR (“SARS Coronavirus 2 Vaccines”)) OR (“COVID-19 Vaccine”))) AND (((((((((“SARS-CoV-2”) OR (“COVID-19”)) OR (”2019-nCoV Disease”)) OR (“COVID19”)) OR (“SARS Coronavirus 2 Infection”)) OR (“Coronavirus”)) OR (“Severe Acute Respiratory Syndrome Coronavirus 2 Infection”)) OR (“COVID-19 Pandemic”))).

### 2.2 Eligibility criteria

This systematic review included all relevant descriptive and observational studies (cross-sectional and longitudinal) on vaccine hesitancy and acceptance. No time constraints for studying or publishing articles nor restrictions on the population were imposed. Non-English studies, studies without full-text access, and those not relevant to vaccine hesitancy or acceptance were excluded. Narrative reviews, systematic reviews, meta-analyses, editorials, commentaries, letters to the editor, unpublished data, books, and conference papers were also excluded.

### 2.3 Study selection

After searching the databases, all retrieved records were screened for inclusion by reviewing the title/abstract and full text based on the eligibility criteria. Six authors (MB, FF, FG, HB, RR, and FS) performed both title/abstract and full-text screening, such that every article was reviewed by two independent reviewers. They resolved any disagreements by consulting a third reviewer (AL, NS, or MA).

### 2.4 Data extraction and analysis

One reviewer (FG, HB, RR, or FS) extracted the relevant data from the included papers, which were then rechecked and confirmed by another reviewer (AL, NS, or MA). The following data were extracted for each study: title, first author's name, date of study (year and month), study design, number of respondents/participants, age groups, gender, race/ethnicity, religion, marital status, country, metropolitan classification (rural or urban), income, insurance status, education, occupation/employment status, work setting (high-risk or non-high-risk), presence of any disease/chronic situation/history of comorbidities (physical/psychiatric), ongoing treatments, smoking status/alcohol consumption, mistrust in the government/healthcare system, received training on COVID-19 prevention, contact with confirmed/suspected COVID-19 patients, history of COVID-19 diagnosis, lost someone from COVID-19, health believes on COVID-19 (perceived susceptibility, severity, benefits, barriers, cues to action, etc.), being informed about COVID-19 vaccines, vaccination-related intentions, parents' willingness and hesitancy toward children's vaccination, COVID-19 vaccination status (not vaccinated, 1 dose, etc.), vaccine hesitancy, willingness to pay for vaccination, people/participants' attitudes and beliefs toward COVID-19 vaccine, fear of vaccination's adverse effects, and acceptance of other vaccines. WebPlotDigitizer version 4.5 (Pacifica, California, USA) was used to extract data from the figures [11].

Hesitancy, the primary outcome of the study, was considered as any reluctance, delay, or doubt in acceptance, and also refusal of the COVID-19 vaccines. Acceptance was defined as already vaccinated or willing to accept COVID-19 vaccines in the future without any doubt. For studies in which only hesitancy or acceptance was reported, the other was calculated by subtracting the total number of respondents from the reported outcome. If a study reported hesitancy and acceptance as two different variables (i.e., measured using two different questionnaires), acceptance was calculated by subtracting the number of hesitant respondents from the total respondents. To measure the effects of age and income on vaccine hesitancy, the highest category of each variable was reported in the included studies and compared with the lowest category. Odds ratio (*OR*) was used to assess the effects of population characteristics on vaccine hesitancy, and event rate (acceptance rate) was the effect measure for overall acceptance. Additionally, 95% confidence intervals (*CIs*) were calculated for both measures. Crude data were extracted when available; otherwise, *ORs* were calculated. Owing to the heterogeneity in the variables included in the regression models of different studies, only univariate *ORs* were extracted for use in the meta-analysis. The random-effects model was used when heterogeneity was more than 50% (I^2^ > 50%). Publication bias was visually assessed using funnel plots and Egger's test. The trim-and-fill method was used to impute the missing studies and adjust for the effects of publication bias.

All meta-analyses were performed using Comprehensive Meta-Analysis, Version 2.2 (CMA; Biostat Inc., Englewood, NJ, USA). The acceptance rate was plotted on the Earth map using Python version 3. The studies were ordered alphabetically in all forest plots.

## 3 Result

### 3.1 Study characteristics

**Fig. 1:**
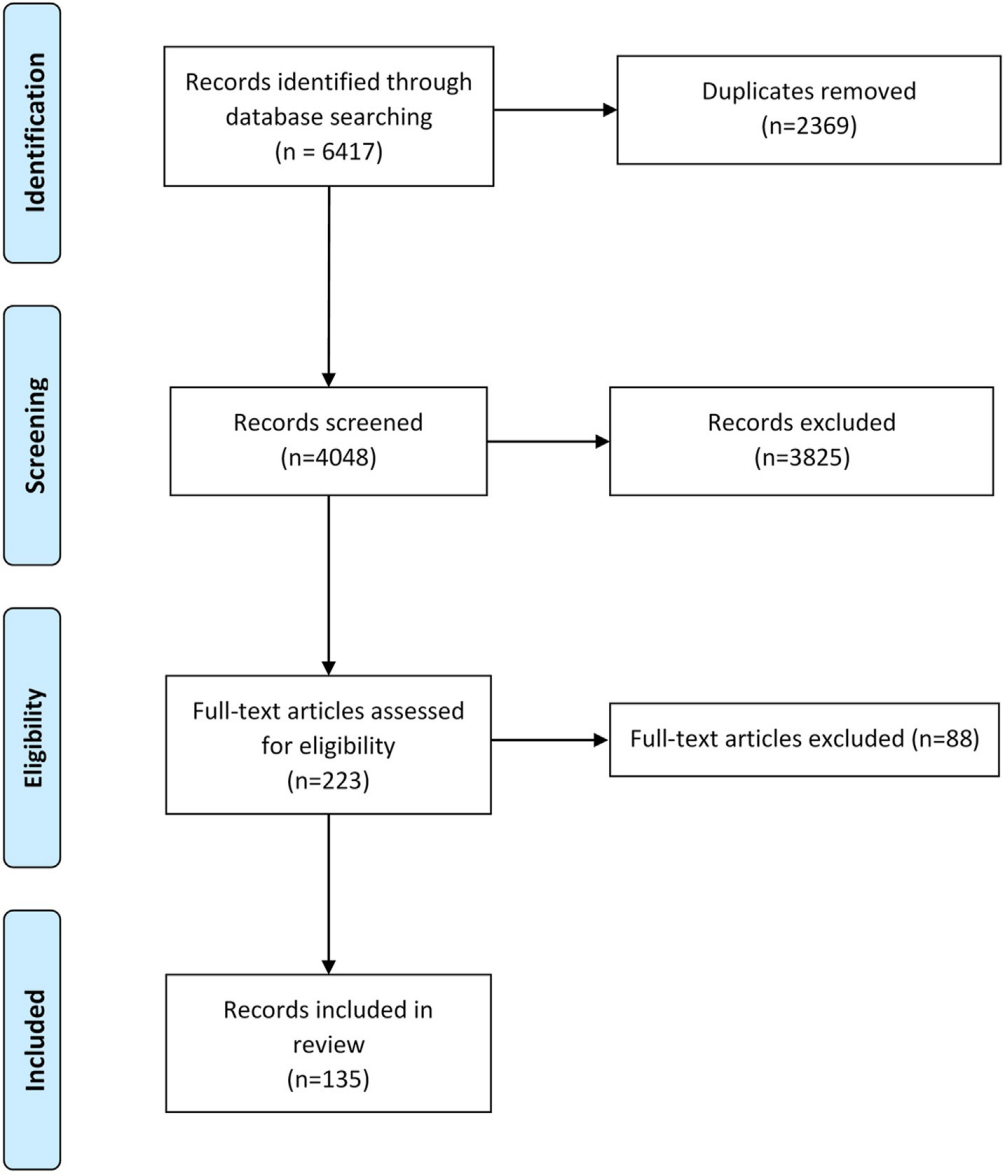
PRISMA 2009 flow diagram.

A total of 6,417 articles (PubMed = 1,548, Web of Science = 1,077, and Scopus = 3,792) were retrieved from the database search. After removing 2,369 duplicated records, 4,048 remained, which were assessed for eligibility using title/abstract and full-text screening. Finally, 135 studies were included in this analysis. Fig. 1 shows the identification process of the included studies according to the PRISMA 2009 flow diagram [12]. Table 1 shows a summary of the included studies.

**Table 1:** Summary of the included articles.

Author	Date	Study design	Region	Population	Number of participants	Factors associated with hesitancy	Factors associated with acceptance
Kwok et al. [13]	Mid-March to late April 2020	Cross-sectional	Hong Kong, China	Nurses	1205	Successful crisis response on the COVID-19 epidemic in Hong Kong, the distrust in health authorities	Younger age, stronger confidence and collective responsibility, insufficient supply of personal protective equipment, involvement in isolated rooms and unfavorable attitudes towards workplace infection control policies, acceptance of flu vaccine
Borriello et al. [14]	27 to 31 March 2020	Cross-sectional	Australia	General	2136	–	–
Mercadante et al. [7]	23 to 29 October 2020	Cross-sectional	US	General	525	Respondents aged 18–49 years old, lower income	Encounter with COVID-19 patients, acceptance of flu vaccine
Wang et al. [15]	26 February to 31 March 2020	Cross-sectional	Hong Kong, China	Nurses	806	Suspicion on efficacy, effectiveness and safety	Aged 30–39, history of chronic disease, encounter with suspected or confirmed COVID-19 patients, higher perceived likelihood of being infected, acceptance of flu vaccine
Mejri et al. [16]	February to May 2021	Cross-sectional	Tunisia	Cancer patients	329	Thinking that the vaccine may interfere with treatment efficacy or may impact cancer outcome, fear of side effects	–
Eyllon et al. [17]	February to May 2021	Cross-sectional	US	Psychiatric patients	14365	Female sex, African Americans, lower income, lower education, tobacco use and previous COVID-19 infection, attention deficit and hyperactivity, posttraumatic stress, bipolar, generalized anxiety, major depressive disorder	Physical co-morbidities
Nguyen et al. [18]	6 January to 29 March 2021	Cross-sectional	US	General	459235	Non-Hispanic blacks, lower income, previous COVID-19 diagnosis	Older than 65
Do et al. [19]	1 June to 28 July 2021	Cross-sectional	US	General	554103	Non-Hispanic Blacks, males, younger age, married, lower income, lower education, having no health insurance, prior COVID-19 infection	–
Kaida et al. [20]	August 2020 to March 2021	Cross-sectional	Canada	General and people living with HIV	5588	Racialized individuals, lower income, lower education, HIV infection	–
Hosokawa et al. [21]	July to August 2021	Cross-sectional	Japan	Pregnant women	1621	Lack of trust in the government, anxiety about potential negative effects on the fetus and breast feeding, adverse reactions at the time of injection	–
Rodriguez et al. [22]	November to December 2020	Cross-sectional	US (Southeast Florida in the Miami region)	People living with HIV	175	Lower education, believing that their health conditions would make it unsafe for them to receive the vaccine or not perceiving their health conditions important for them to receive the vaccine	–
Cáceres et al. [23]	February 2021	Qualitative research	US	Community health workers	22	Misinformation, mistrust in the government and the scientific community, members of Latino community	–
Lin et al. [1]	1 to 19 May 2020	Cross-sectional	China	General	3541	Concerns about safety and efficacy, lower income	–
Delgado-Gallegos et al. (A) [24]	December 2020 to February 2021	Cross-sectional	Mexico	Parent's perspective towards child COVID-19 vaccination	699	Younger parents, fear of adverse effects	Parents believing that the COVID-19 vaccine will enhance the economic situation of the country, parents actively researching information, having the willingness to obtain the COVID-19 vaccine themselves, acceptance of flu vaccine
Graffigna et al. [25]	During the early days of the Italian reopening after the lockdown	Cross-sectional	Italy	–	1004	–	Perceived severity, perceived susceptibility, general attitude towards a vaccine
Kreps et al. [26]	9 July 2020	Cross-sectional	US	–	1971	Fear of adverse effects, FDA emergency use authorization	–
Iadarola et al. [27]	A year-long statewide campaign from December 2020	Cross-sectional	US	Intellectual and developmental disabilities community	825	Black and Hispanic, younger age, not trusting the government, concerns about side effects	–
Willis et al. [28]	22 April to 6 July 2021	Cross-sectional	US	General	1475	Black/African Americans	Older age, men, less than high school degree, not having health insurance, acceptance of flu vaccine
Zhang et al. [29]	4 to 13 August 2020	Mixed method (cross-sectional and qualitative)	China	General	19132	self-conceit, confidence, accessibility	Knowledge, information attention and trust in official media, perceived susceptibility and controllability, preventive behaviors
Caserotti et al. [30]	January to February 2021	Cross-sectional	Italy	General	5006	Females, younger age, high level of conspiracy-mindedness	Higher education, chronic diseases, knowing someone who died from COVID-19, general acceptance of vaccines
Marzo et al. (A) [31]	February to May 2021	Cross-sectional	Indonesia, Malaysia, Myanmar, Philippines, Thailand, Vietnam	General	5260	Low and high economic status, living in rural areas, lower education, unemployed	Males, never married
Marzo et al. (B) [32]	February to May 2021	Cross-sectional	20 countries across four continents	General	10477	Age groups of 31–45 and 46 to 60, unmarried individuals, low-economic families	Male, residing in urban areas, higher education, being student, unemployed individuals, medium- and high-economic families
Cook et al. [6]	January to March 2021	Cross-sectional	UK	General	1058	Black Africans, age under 30	Presence of chronic health condition
Paul et al. [8]	October 2020	Qualitative research	Austria, Belgium, Germany, Ireland, Italy, German-speaking Switzerland, UK	General	246	–	–
Dobbs et al. [33]	26 January to 17 March 2021	Cross-sectional	Puerto Rico	General	173	30-65 years old, lower education, low general vaccine confidence	18–29 years old
Liao et al. [34]	June to November 2020	Six rounds of cross-sectional survey	Hong Kong, China	General	4055	18-34 years old, lowest and highest educational attainment, low general vaccine confidence	>35 years old, perceived greater severity
Palamenghi et al. [35]	During the early days after the initial spread of the SARS-CoV-2 virus in Italy (phase 1) and during the early days of the Italian reopening after lockdown (phase 2)	Cross- sectional in 2 Phases	Italy	General	The first survey: 968/the second: 1004	The middle-age group had a reduced willingness to vaccinate compared to the 18–34 years old	Trust in scientific research and general attitude towards vaccines' efficacy
Jan et al. [36]	10 to 20 May 2021	Cross-sectional	Saudi Arabia	Sickle cell disease patients	147	Fear of developing consequences and brain blood clots	–
Zhang et al. [4]	16 to 20 August 2021	Cross-sectional	China	Health care students	631	–	Greater perceived severity
Hulen et al. [37]	6 to 21 July 2021	Cross-sectional	US	Veteran's affairs health care system employees	1157	Fear of adverse reactions, not knowing anyone who died from COVID-19	–
Bateman et al. [38]	December 2020	Qualitative research	–	–	67	Mistrust, fear, and lack of information	–
Nery et al. [3]	November 2020 to January 2021	Cross-sectional	Brazil	–	2521	–	Older age, white, presence of comorbidities, male, employed, higher education, having previous symptoms compatible with COVID-19 and report of previous testing for COVID-19, higher perceived risk, acceptance of flu vaccination
Elliott et al. [39]	February to March 2021	Cross-sectional	US	College students	3773	Aged 46–50, American Indians and Blacks, being suspicious of vaccines in general, fear of side effects	Married or living with a partner, living in large cities, higher education
Lee et al. [40]	20 January 2021	Cross-sectional	South Korea	General	1016	Females, unmarried, seeking COVID-19 vaccine-related information via social medias, mistrust in government	Presence of underlying disease, acceptance of flu vaccine, higher perceived risk
Harada et al. [41]	September to April 2021	Cross-sectional	Japan	General	1000	Lower education, mistrust in government	–
Duong et al. [42]	May 2021	Qualitative research	Vietnam	General	20	Mistrust in government	Higher vaccine knowledge
Toth-Manikowski et al. [43]	March to May 2021	Cross-sectional	US	Health care workers	1974	18-44 years old, women, black and African Americans, fear of adverse effects	–
Kuhn et al. [44]	December 2020 to January 2021	Longitudinal study	US	Homeless in Los Angeles	3145	Ages 43.4–51.7, blacks, fear of side effects	–
Alshahrani et al. [45]	January 2021	Cross-sectional	Saudi Arabia	General	758	–	Males, university level graduates, acceptance of flu vaccine
Thunström et al. [46]	March 2020	Cross-sectional	US	General	3133	Females, mistrust in government, fear of side effects	Acceptance of flu vaccine
Fedele et al. [47]	14 to 28 November 2020	Cross-sectional	Italy	General	2260 families	Unemployed, lower education	>35 years
Guljaš et al. [48]	December 2020 to January 2021	Cross-sectional	Croatia	General	276	Younger than 40	Health workers
Abu Farha et al. [49]	July to August 2020	Cross-sectional	Jordan	General	1278	Religious/cultural beliefs, non-married, lower education	–
Piltch-Loeb et al. [50]	13 to 23 December 2020	Cross-sectional	US	General	2650	–	–
Urrunaga-Pastor et al. [51]	15 January to 1 February 2021	Cross-sectional	Latin America and the Caribbean	General	472521	Non binary group, rural areas, economic insecurity, no COVID-19 symptomatology, fear of adverse effects	–
Bass et al. [52]	15 May to 6 July 2020	Cross-sectional	US	General	501	Females, blacks, less income, less education	–
Chew et al. [53]	12 to 21 December 2020	Cross-sectional	Asia–Pacific, China	General	1720	Previous heart failure, undergraduate	–
Fridman et al. [54]	16 March to 16 August 2020	Longitudinal study	US	General	The first survey: 1018/the next surveys: 608–762	–	–
Latkin et al. [55]	The first survey: 24 to 27 March/the second:5 to 14 May/the third survey: 22 to 30 July	Longitudinal study	US	General	592	Females, blacks, lower income, lower education, better health status, high trust in white house and very conservative	–
Vallée et al. [56]	January 2021	Cross-sectional	France	People living with HIV	237	–	–
Reno et al. [57]	January 2021	Cross-sectional	Italy	General	1011	Aged 35–54, females, low income, low education, absence of comorbidities	–
Saied et al. [58]	January 2021	Cross-sectional	Egypt	Medical students	2133	Females, good Self-perception of own health, no previous COVID-19 infection, concerns about adverse effects	Acceptance of flu vaccine
Soares et al. [59]	29 September 2020 to January 2021	Open cohort	Portugal	General	1943	Females, lower income, university education, very good/good perception of health, no/low/Moderate Self-perceived risk to get COVID-19 infection	Acceptance of flu vaccine
Caserotti et al. [60]	February to June 2020	Cross-sectional	Italy	General	2267	–	<25 years old
Mena et al. [61]	21 December 2020 to 4 January 2021	Cross-sectional	Spain	Hospital staff	906	Females, younger age, less education, auxiliary nurses	Physicians, no underlying disease, having close contact occupation, flu vaccine acceptance
Alhassan et al. [62]	18 September to 23 October 2020	Cross-sectional	Ghana	General	1556	Males, aged 18–47, Christians, unmarried, lower education	–
Wang J et al. [63]	November 2020 to January 2021	Cross-sectional	China	Elderly and chronic disease population	7259	Older age, mistrust in government, concern about vaccine safety, low infection risk, waiting and seeing others getting vaccinated, concern about vaccine effectiveness and price	–
Wu et al. [64]	6 to 9 August 2021	Cross-sectional	China	General	29925	Men, religious, >60 years old, unmarried, less education, presence of chronic disease, current smoker, former drinker, mistrust in health care system	–
Zhang X et al. [65]	17 April to 28 May 2021	Cross-sectional	–	General	1015	Aged 30–39, lower education, lower income	–
Samannodi et al. [66]	6 June to 9 July 2021	Cross-sectional	Saudi Arabia	Care givers (willingness to vaccinate their children)	581	–	–
Wong et al. [67]	23 April to 8 May 2021	Cross-sectional	Hong Kong, China	General	1195	–	Older age
Dye et al. [68]	6 April to 29 May 2020	Cross-sectional	173 countries	General	7411	Resided in Africa, direct COVID-19-related experience (had a family or friend die from COVID-19, or believed they have COVID-19 themselves)	<32 years old, females, higher education, chronic disease, higher COVID-19 related knowledge, adherence to preventive strategies
Fernández-Penny et al. [69]	29 January to 11 May 2021	Cross-sectional	US	Patients in two urban emergency departments	1068	<35 years old, female, black, on government insurance plans, lower education, mistrust in government	On private insurance
Gatto et al. [70]	15 March to 26 April 2021	Cross-sectional	US	Employees of a Safety Net California County Health System	789	30-49 years old, females, lower income, lower education, nurses, nursing assistants and medical assistants, administrative, non-clinical staff, asthmatic	Acceptance of flu vaccine
Amuzie et al. [71]	12 May 2021	Cross-sectional	Nigeria	Health care workers	422	Younger age, single, lower income, non-clinical staff	Doctors, nurses, allied health professionals,
Iliyasu et al. [72]	March 2021	Mixed-methods design	Nigeria	General	446	Worried about safety and rumors,	Chronic medical disorder, higher risk perception, higher income, >30 years old
Liddell et al. [73]	13 October 2021	Cross-sectional	Australia	Refugees	516	–	>60 years old, higher risk perception
Bolatov et al. [74]	March 2021	Cross-sectional	Kazakhstan	Students at Astana Medical University	888	–	Contextual effects (for instance, communication and social media, socio-demographic factors, vaccination policies, perception of the pharmaceutical industry), individual and group effects (for instance, personal experience with vaccination, attitudes about public health and prevention, trust in the health system, perceived risk), specific issues on COVID-19 vaccination (for instance, risk/benefit, source of supply of vaccine, knowledge base on vaccination)
Montalti et al. [75]	December 2020 to February 2021	Cross-sectional	Italy	General	443	>40 years old, females, lower education, worries about safety, mistrust in health care system, lack of information	Acceptance of flu vaccine
Umakanthan et al. [76]	4 August 2021	Longitudinal study	India	General	3000	Worries about side effects and contradiction with chronic diseases	Trust in government and health care system, more sense of social distancing, >55 years old, male, higher education
Stead et al. [77]	January to February 2021	Cross-sectional	UK	General	4978	–	Older age, white, higher education
de Sousa et al. [78]	1 September 2021	Observational analytical study	Portuguese-Speaking Countries	General	6843	Older age, female, higher education, close contact with someone who had COVID-19 or died from COVID-19 or tested positive themselves	–
Riad et al. [79]	2 July 2021	Cross-sectional	Czech Republic	Students	1351	Worried about vaccine safety	Trusted the pharmaceutical industry, previous COVID-19 infection, knowing someone who died from COVID-19, acceptance of flu vaccine
Almalki et al. [80]	2 to 23 April 2021	Cross-sectional	Saudi Arabia	University students	407	Fear of side effects	Acceptance of flu vaccine
Xu et al. [81]	19 April 2021	Cross-sectional	China	Parents of primary and middle school students (willingness to vaccinate their children)	4748	Worries about safety, females, psychological distress	–
Adams et al. [82]	3 May 2021	Cross-sectional	US	Young adults	5082	Wait and see if the vaccine is safe, concerns over side effects, believing others are in greater need of the vaccine	–
Mant et al. [83]	The first survey: 20 June to 28 July 2020/the second: 22 September to 17 October 2020	A multi-methods study	Canada	University students	The first survey: 483/the second survey:1269	–	Being encouraged by their doctor or pharmacist, higher severity perception, being affected by COVID-19
Baniak et al. [84]	February 2021	Cross-sectional	US	Nursing staff	276	Worries about side effects	Greater than 10 years' work experience, confidence in vaccine safety
Khubchandani et al. [85]	10 December 2020	Cross-sectional	US	General	1878	African Americans, Hispanic, females	Higher education, higher income
Rozek et al. [86]	May to June 2020	Cross-sectional	17 countries	General	–	–	Older age, males, higher income, not trusting religious leaders, trust in science
Jain et al. [87]	2 February to 7 March 2021	Cross-sectional	India	Medical students	1068	Obtaining information from social media, concern regarding vaccine safety and efficacy and lack of trust in public health authorities	–
Afifi et al. [88]	26 March 2021	Observational longitudinal study	Canada	General	664	Lower income, concern regarding vaccine safety and efficacy and lack of knowledge	–
Castañeda-Vasquez et al. [89]	October to December 2020	Cross-sectional	Mexico	Health personnel	543	upper-middle/upper economic class, not belonging to medical guide, having children, vaccine related misinformation	–
Anjorin et al. [90]	25 April 2021	Cross-sectional	African countries	General	5416	Worries about side effects, females	Urban, employed, higher risk perception, general vaccination acceptance and knowledge
Griva et al. [91]	June to July 2021	Cross-sectional	Singapore	Hesitancy for adults and children in the Singapore Population	1623	Fears about side effects and safety, employed, lower risk perception, female, 31–40 years old	–
AlSaeed et al. [92]	16 October 2021	Cross-sectional	Saudi Arabia	General	486	Worries about effectiveness, conspiracy theories	–
Khidir et al. [93]	9 to 16 May 2021	Cross-sectional	Kurdistan Iraq	General	450	Low risk perception, unemployed, 26–40 years old, lower education	
Mahmud et al. [94]	30 January to 6 February 2021	Cross-sectional	Bangladesh	General	605	Worries about side effects and safety	>30 years old, males, urban, married, higher education, higher risk perception, knowledge about COVID-19, acceptance of other vaccines
Alibrahim et al. [95]	22 August 2021	Cross-sectional	Kuwait	General	4147	Females, married or divorced, mistrust in health care system, worries about safety and side effects	Acceptance of flu vaccine
Wang K et al. [96]	26 to 28 February 2021	Cross-sectional	Hong Kong, China	Working-age People	1773	Vaccination at health care facilities, exposure to vaccination information from social media	–
Allington et al. [97]	1 April 2021	Cross-sectional	UK	General	4343	Low risk perception, low trust in science, lower education, lower income, younger age	–
Schernhammer et al. [98]	5 May 2021	Cross-sectional	Austria	General	1007	Younger age, females, rural areas, mistrust in government or did not vote in the last election	–
Nath et al. [99]	29 November 2021	Cross-sectional	Bangladesh	General	343	Conspiracy theories	–
Harapan et al. [100]	20 December 2021	Cross-sectional	10 countries in Asia, Africa, South America	General	1832	Females	Acceptance of flu vaccination, health care workers
Willis et al. [101]	29 September 2021	Cross-sectional	US	Youth	345	Watching TV, playing video games	Acceptance of flu vaccination
Gerretsen et al. [102]	May to July 2020/March 2021	Cross-sectional	US and Canada	General	7678	Younger, females, Blacks, rural, lower income and education, employed, right wing political status	Retired, more health risk factors, higher risk perception
Konstantinou et al. [103]	31 March 2020 to 17 March 2021	Cross-sectional	Cyprus	General	701	–	Higher risk perception, higher education, females, older age
Bronstein et al. [5]	1 to 8 April 2021	Cross-sectional	US	–	554	Less data gathering, conspiracy theories	Higher risk perception,
Yanto et al. [104]	15 to 25 March 2021	Cross-sectional	Indonesia	General	190	More frequently tested for COVID-19, smokers, trust in government, higher education, higher income, no insurance, unmarried, men	–
Andrade (A) et al. [105]	February to May 2021	Cross-sectional	Venezuela	University students	230	Religiosity	–
Carson et al. [106]	16 November 2020 to 28 January 2021	Original study (qualitative)	US	Minorities	70	Political issues, unreliable information, not paid time off	–
Corcoran et al. [107]	17 May to 1 June 2021	Cross-sectional	US	General	2003	Less income, pro trump ideology, younger, religious	–
Andrade (B) et al. [108]	February 2021	Cross-sectional	Venezuela	University students	273	Marginally ethnic group	–
Hossain et al. [109]	22 March to 1 April 2021	Cross-sectional	Bangladesh	University students	900	Females, rural, lower income and lower education, no history of COVID-19, inadequate knowledge, worries about safety, efficacy and side effects	–
Delgado-Gallegos (B) et al. [110]	December 2020 to February 2021	Cross-sectional	Mexico	General	1481	Unemployed, no history of COVID-19, lower education, religious, 18–24 years old	Research about vaccines, acceptance of flu vaccine
Liu et al. [111]	4 April to 24 May 2021	Cross-sectional (qualitative)	China	–	7379	Living and working in less populated areas, adverse side effects, chronic diseases like eczema	–
Hwang et al. [112]	October to December 2020	Cross-sectional	South Korea	General	13353	Religious, low income, unstable job, unmet medical needs, political conservatism, worries about safety	–
Muhajarine et al. [113]	4 May 2020 to 3 April 2021	Cross-sectional	Canada	General	9252	Good health, indigenous, financially insecure, females, living alone	Higher risk perception, more contact with others, higher education
Bacon et al. [114]	16 October 2020 to 15 January 2021	Cross-sectional	UK	General	208	Mistrust in pharmaceutical companies, mistrusting vaccines in general, worries about side effects	–
Sallam et al. [115]	14 to 18 December 2020	Cross-sectional	Arabic countries	General	3414	Against vaccines in general, relying on social media, history of COVID-19, lower education and income, females	–
Yoda et al. [116]	September 2020	Cross-sectional	Japan	General	1100	Concerns about side effects	Older age, males, rural, chronic disease, acceptance of flu vaccine
Faezi et al. [117]	15 February to 15 April 2021	Cross-sectional	Differed countries mostly Iran	General	1880	–	Males, 20–60 years old, without background disease, acceptance of other vaccines, higher education, trust in government
Gendler et al. [118]	June 2021	Cross-sectional	Israel	Parents (willingness to vaccinate their children)	520	–	Higher vaccine literacy
Cordina et al. [119]	The first survey: 30 October to 11 December 2020/the second: 26 October to 26 December 2020	Cross-sectional	International	General	2529 first study, 843 s study	–	>60 years old, males, acceptance of flu vaccine, trusting health professionals
Berry et al. [120]	30 December 2020 to 15 January 2021	Cross-sectional (qualitative)	US	Skilled nursing facility	193	Concerns about side effects, blacks indigenous and people of color	–
Kreps et al. [121]	29 to 30 October 2020	Cross-sectional	US	General	1096	–	Older, higher income, higher education, past flu vaccination, informed about the vaccine
Ebrahimi et al. [122]	23 January to 2 February2021	Cross-sectional	Norway	General	4571	Having children, trust in social media, rural areas, females	Health sector employees
Savoia et al. [123]	13 to 23 December 2020	Cross-sectional	US states and the territories of Puerto Rico, American Samoa, Guam	General	2560	Younger age, males, blacks, history of severe COVID-19	3 or more medical conditions, higher education
Al-Mohaithef et al. [124]	January to March 2021	Cross-sectional	Saudi Arabia	General	658	–	Higher risk perception, trust in health care system, married
Walker et al. [125]	March to May 2020	Cross-sectional (qualitative)	US	Mothers (willingness to vaccinate their children)	25	Safety, skepticism about efficacy, feeling rushed, confusion	–
King et al. [126]	18 February to 17 March 2021	Cross-sectional	Austria	General	1350	Lower income and education	Older
Jin et al. [127]	May 2021	Cross-sectional	Pakistan	General	320	–	Higher perceived risk
Al-Sanafi et al. [128]	18 to 29 March 2021	Cross-sectional	Kuwait	Health care workers	1019	–	Males, higher education, health care workers
Jiang et al. [129]	1 March to 30 June 2020	Cross-sectional	US		5000 tweets	Conservatives	
Alzahrani et al. [130]	25 December 2020 to 15 February 2021	Cross-sectional	Saudi Arabia	General	3048	Females, lower income and higher education, lower risk factors, concerns about safety and side effects, not vaccinated against flu	–
Magadmi et al. [131]	May 2020	Cross-sectional	Saudi Arabia	General	3101	–	Flu vaccine acceptance, higher education, males, <30 years old
Solís Arce et al. [132]	Second half of 2020	Cross-sectional	LMIC	General	44260	Females, concerns about efficacy and side effects	–
Shen et al. [133]	February 2021	Cross-sectional	China	General	2361	–	–
Velikonja et al. [134]	12 February to 5 March 2021	Cross-sectional	Slovenia, Poland and Serbia	Nursing students	872	Fear of side effects, against vaccination in general	Males, health care workers, trust in institutions, previous COVID-19 diagnosis
Chaudhary et al. [135]	January to March 2021	Cross-sectional	Pakistan	General	410	Concerns about safety	Healthier, higher vaccine knowledge, higher education, higher income
Turhan et al. [136]	December 2020	Cross-sectional	Turkey	General	620	Young, females, married, less education, unemployed, distrust in health system,	–
El-Far Cardo et al. [137]	August to November 2020	Cross-sectional	Germany	General	808	–	Males, left wing voters, 70–79 years old, history of COVID-19
Stojanovic et al. [138]	March 2020 to January 2021	Cross-sectional	Brazil, Canada, Colombia, France, Italy, Turkey, UK, US	General	32028	Lower income, lower education, rural areas, unemployed	Males, older, having chronic health conditions, acceptance of flu vaccine
Woolf et al. [139]	December 2020 to March 2021	Cohort	UK	Health care workers	11584	Females, blacks, Nursing, Nursing associates and Midwives, history of COVID-19, conspiracy theories	Doctors and medical support groups, acceptance of COVID-19 vaccine
Leung et al. [140]	Mid-March to April 2020	Secondary analysis	Hong Kong, China	Nurses	1193	–	Higher perceived risk, older, males

COVID-19 = coronavirus disease 2019; HIV = human immunodeficiency virus; UK = United Kingdom; US = United States; LMIC = Lower- and Middle-Income Countries; SNF = Skilled Nursing Facility; TV = Television; FDA = Food and Drug Administration.

### 3.2 ***Results of analysis***

**Fig. 2:**
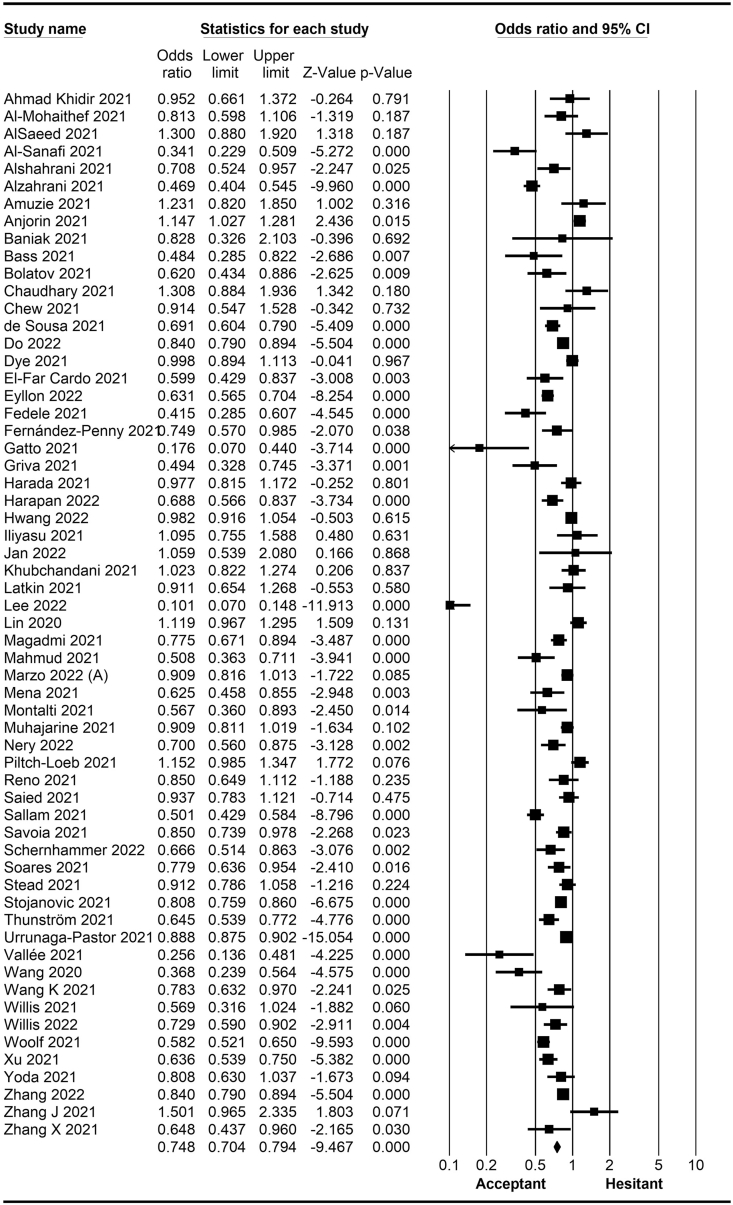
Forest plot displaying the comparison of COVID-19 vaccine hesitancy between women and men.

**Fig. 3:**
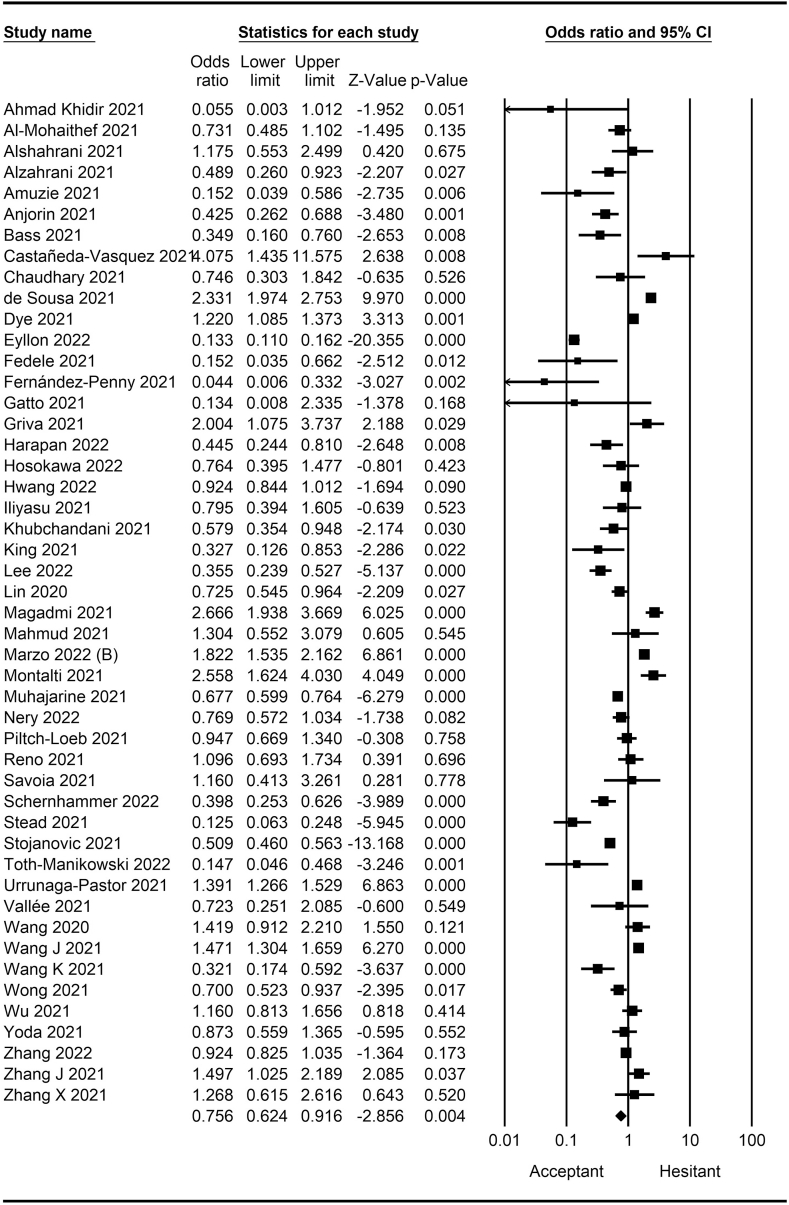
Forest plot displaying the comparison of COVID-19 vaccine hesitancy between older and younger people.

**Fig. 4:**
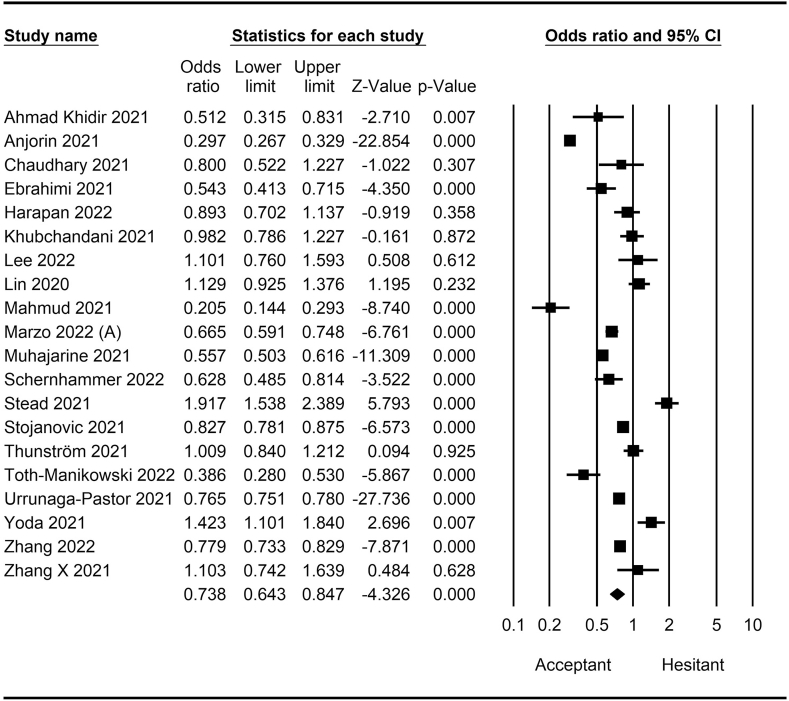
Forest plot displaying the comparison of COVID-19 vaccine hesitancy between urban and rural people.

**Fig. 5:**
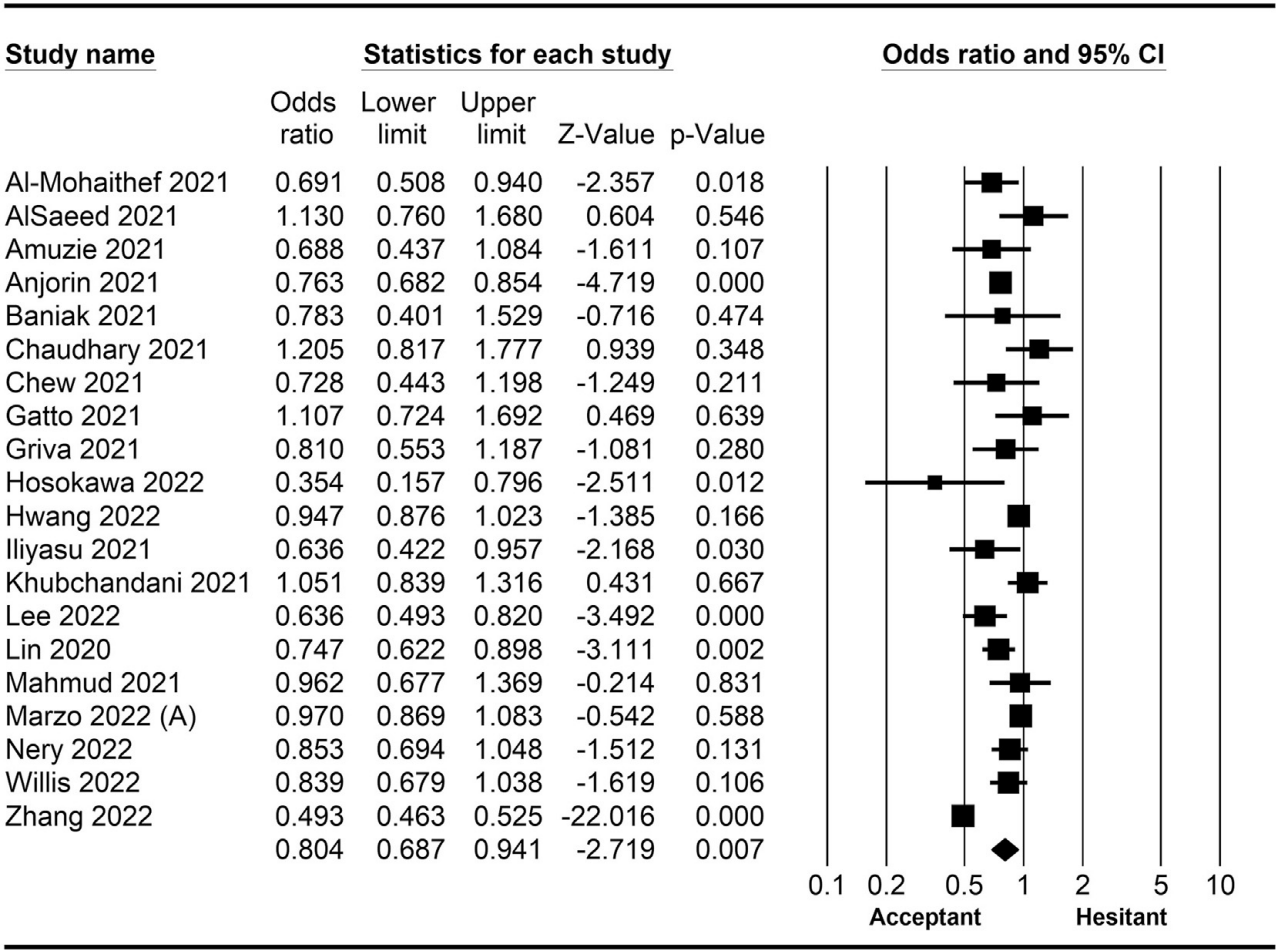
Forest plot displaying the comparison of COVID-19 vaccine hesitancy between married and single people.

**Fig. 6:**
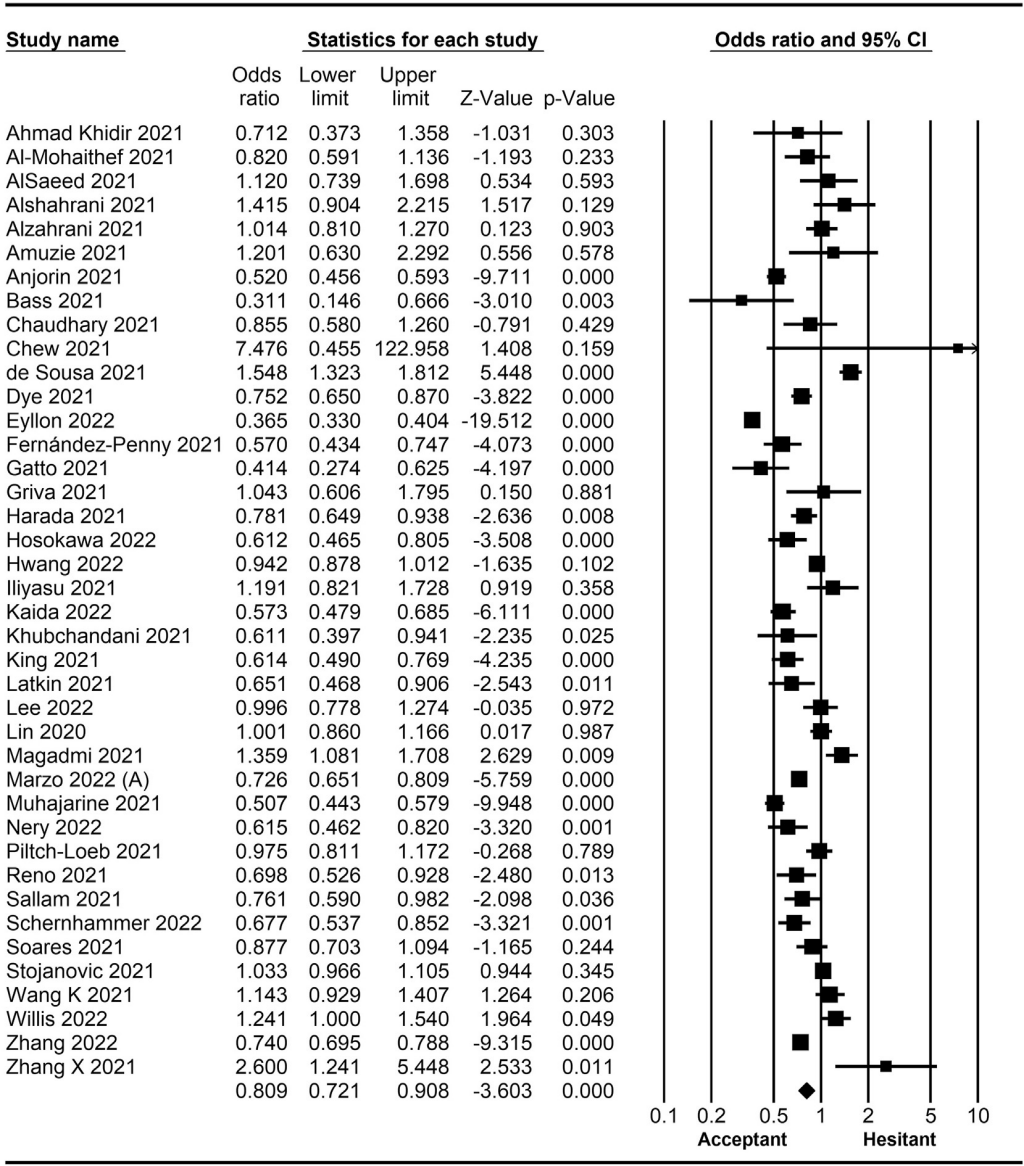
Forest plot displaying the comparison of COVID-19 vaccine hesitancy between educated people and non-educated people.

**Fig. 7:**
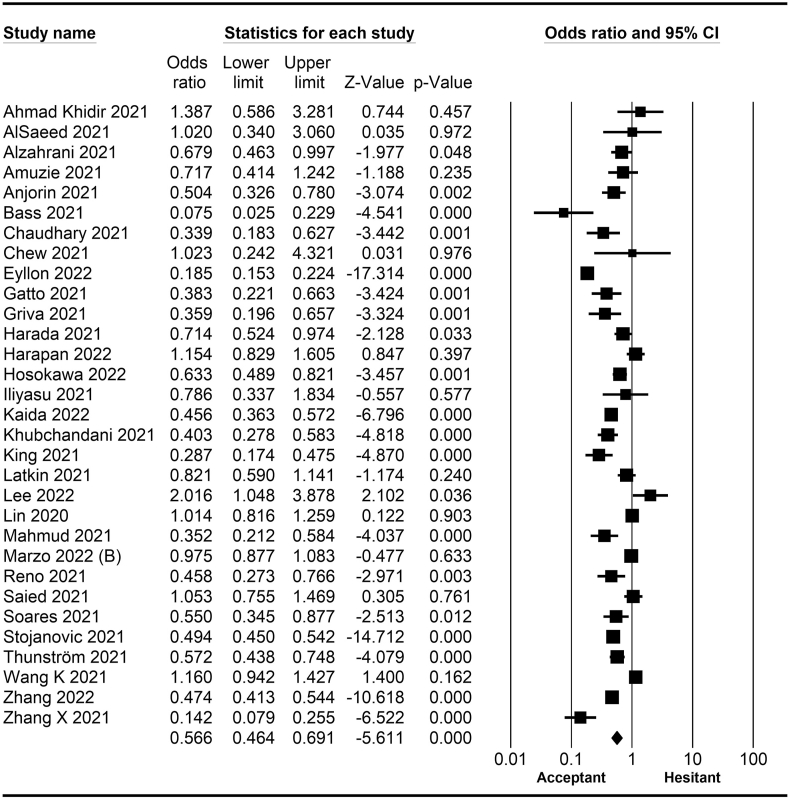
Forest plot displaying the comparison of COVID-19 vaccine hesitancy between high and low-income people.

**Fig. 8:**
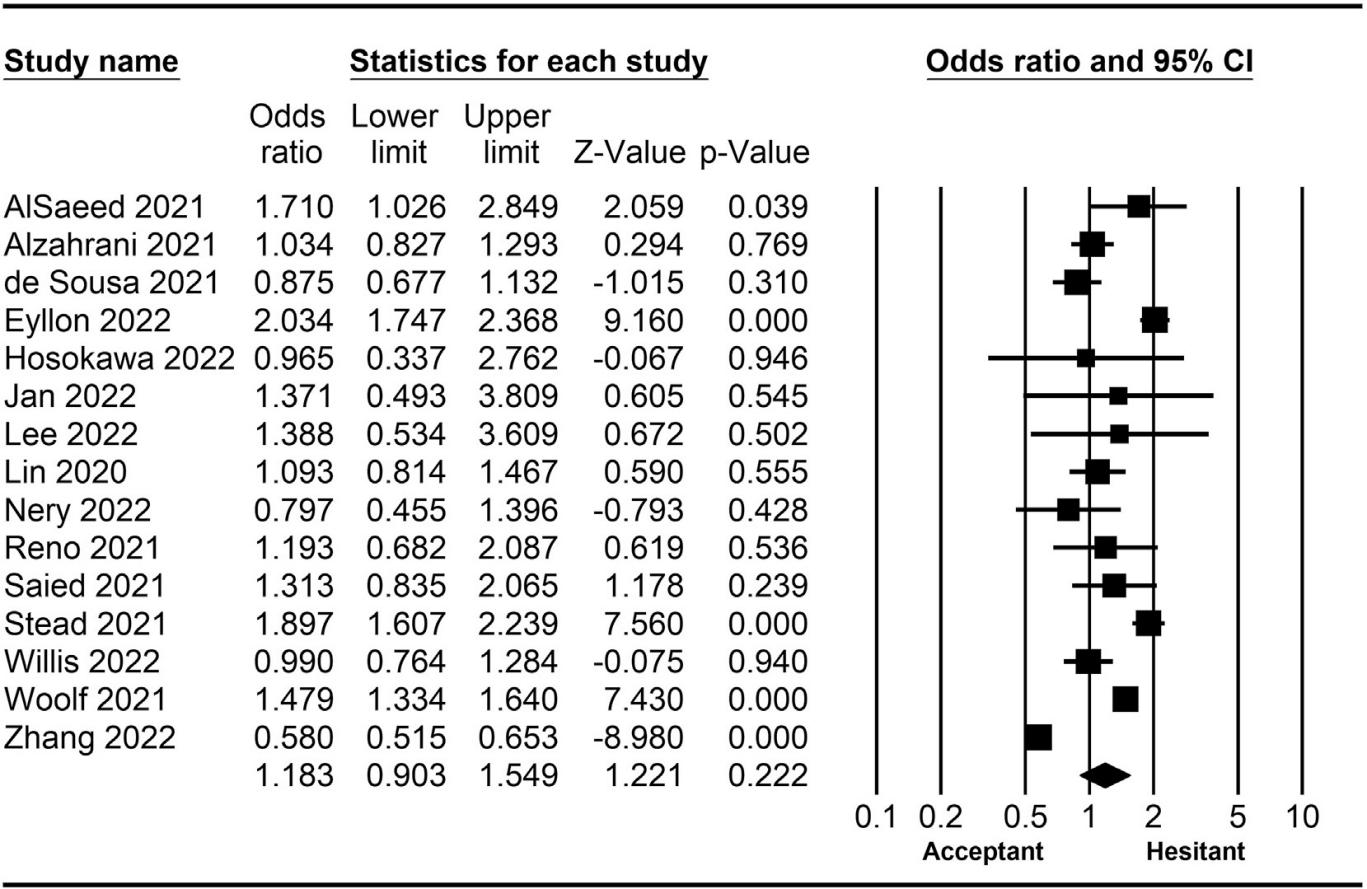
Forest plot displaying the comparison of COVID-19 vaccine hesitancy between people with and without a history of COVID-19 infection.

**Fig. 9:**
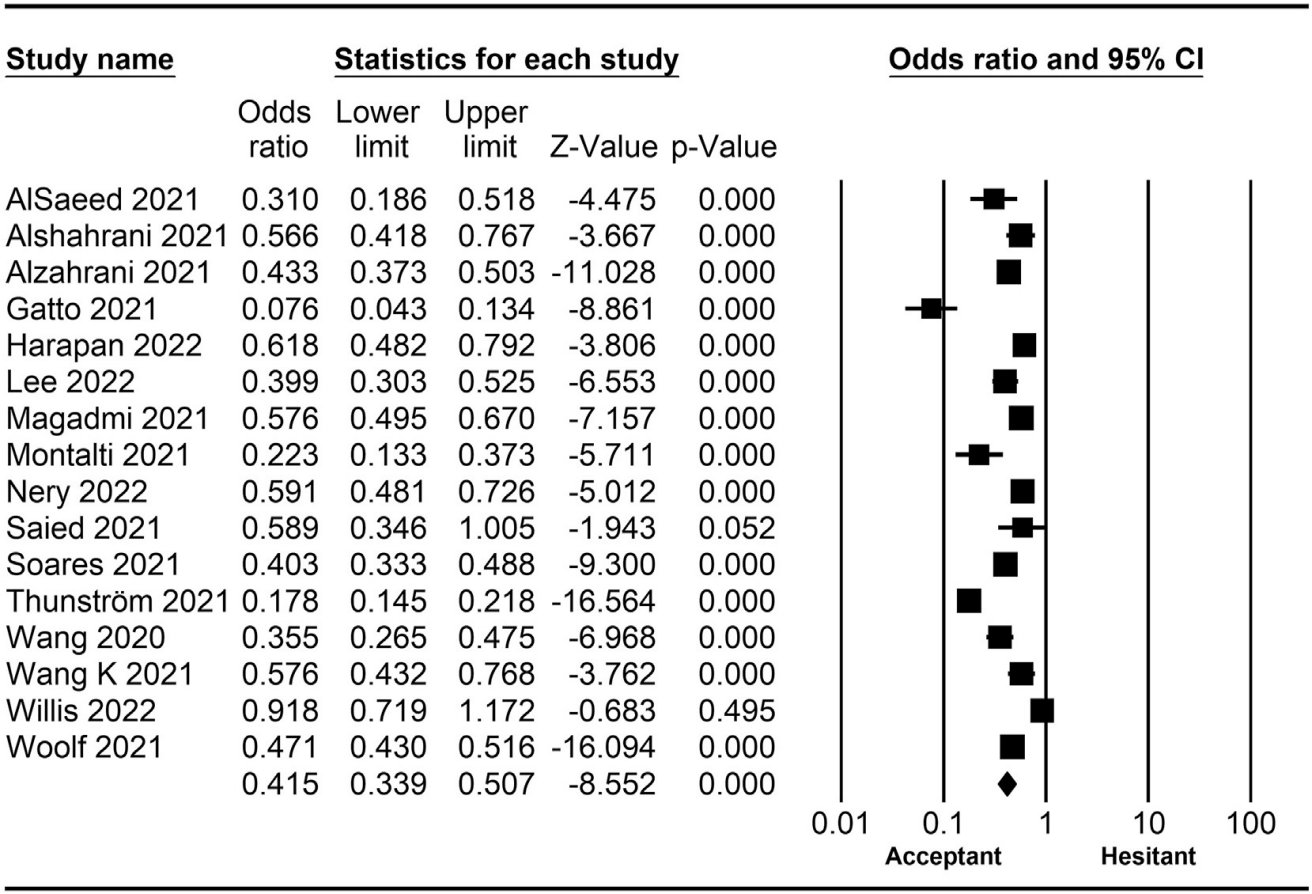
Forest plot displaying the comparison of COVID-19 vaccine hesitancy between people with and without a history of influenza vaccine.

**Fig. 10:**
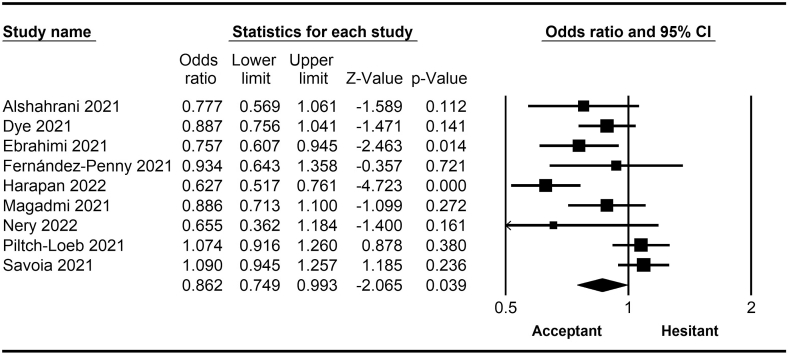
Forest plot displaying the comparison of COVID-19 vaccine hesitancy between healthcare and non-healthcare workers.

**Fig. 11:**
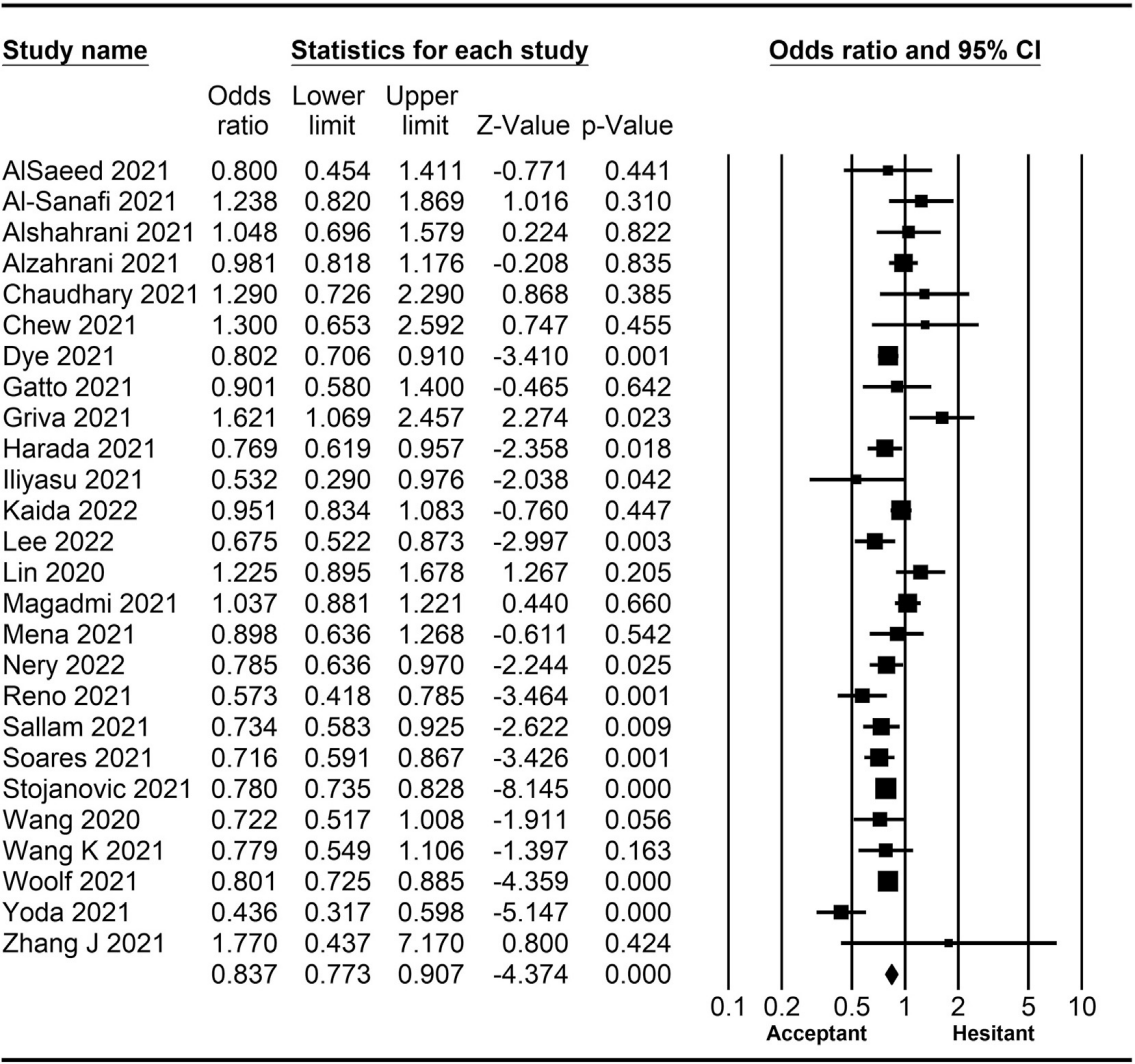
Forest plot displaying the comparison of COVID-19 vaccine hesitancy between people with and without comorbidities.

The rate of COVID-19 vaccine hesitancy differs by gender. Data from 60 studies have shown that the men have a significantly lower rate of vaccine hesitancy compared to women (*OR* = 0.75; 95% *CI* = 0.70–0.79; Fig. 2). Moreover, data from 48 studies have shown that older people tend to have lower vaccine hesitancy than younger people (*OR* = 0.76; 95% *CI* = 0.62–0.92; Fig. 3). The urban population had less vaccine hesitancy compared to the rural population, according to 20 studies (*OR* = 0.74; 95% *CI* = 0.64–0.85; Fig. 4). The proportion of COVID-19 vaccine hesitancy by marital status from 20 studies demonstrated that married people tend to be less vaccine-hesitant compared to single ones (*OR* = 0.80; 95% *CI* = 0.69–0.94; Fig. 5). Data from 40 studies reported that educated people (above high school) showed a significantly lower COVID-19 vaccine hesitancy compared to non-educated people (high school or lower; *OR* = 0.81; 95% *CI* = 0.72–0.91; Fig. 6). Regarding people's financial status, data from 31 studies showed that the high-income population had lower COVID-19 vaccine hesitancy compared to the low-income population (*OR* = 0.57; 95% *CI* *=* 0.46–0.69; Fig. 7). A meta-analysis of 15 studies revealed that people with a history of COVID-19 infection had no statistically significant difference in COVID-19 vaccine hesitancy compared to people without a history of COVID-19 infection (*OR* = 1.18; 95% *CI* *=* 0.90–1.55; Fig. 8). Further, an analysis of 16 studies showed that people with a history of influenza vaccination had lower COVID-19 vaccine hesitancy than people without a history of influenza vaccination (*OR* = 0.41; 95% *CI* *=* 0.34–0.51; Fig. 9). Healthcare workers showed less vaccine hesitancy compared to non-healthcare workers, according to nine studies (*OR* = 0.86; 95% *CI* *=* 0.75–0.99; Fig. 10). Finally, having one or more comorbidities was associated with lower vaccine hesitancy according to 26 studies (*OR* = 0.84; 95% *CI* *=* 0.77–0.91; Fig. 11).

**Fig. 12:**
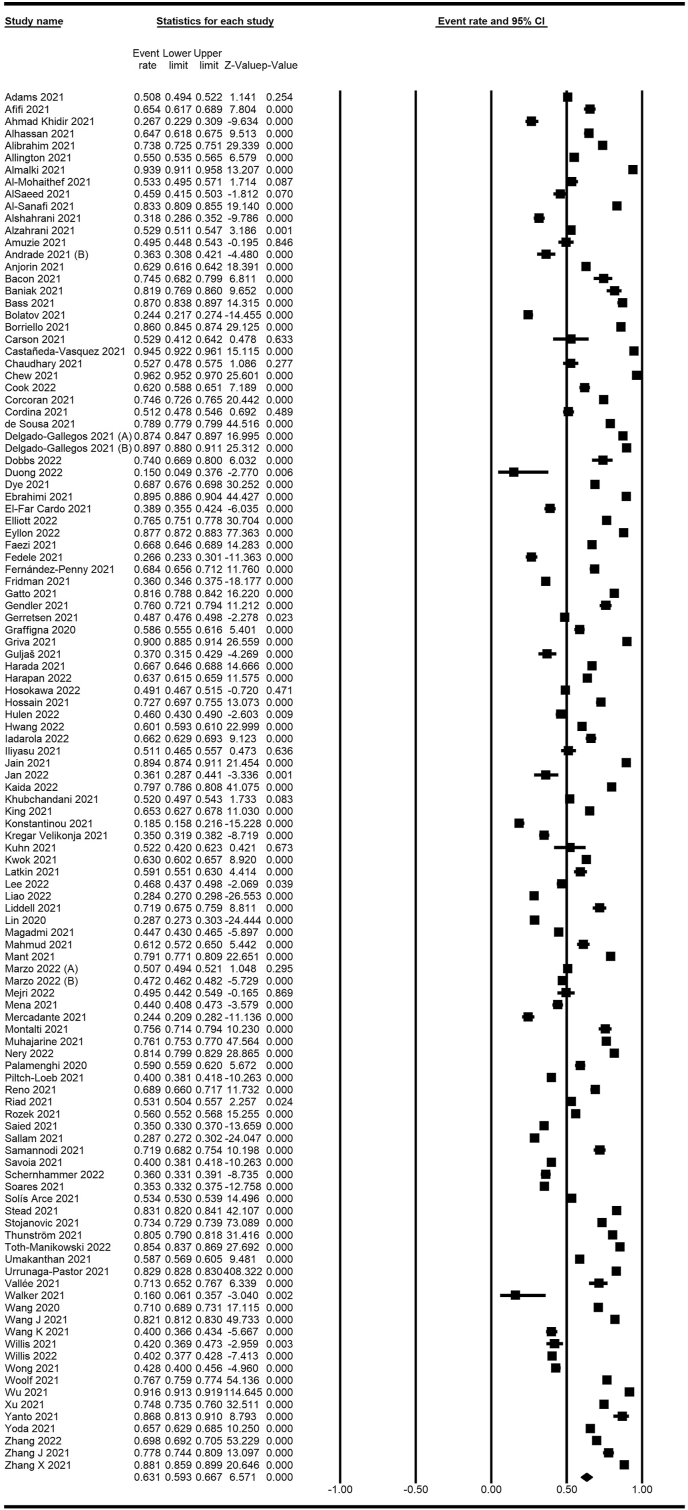
Forest plot displaying the overall acceptance rate of included studies.

**Fig. 13:**
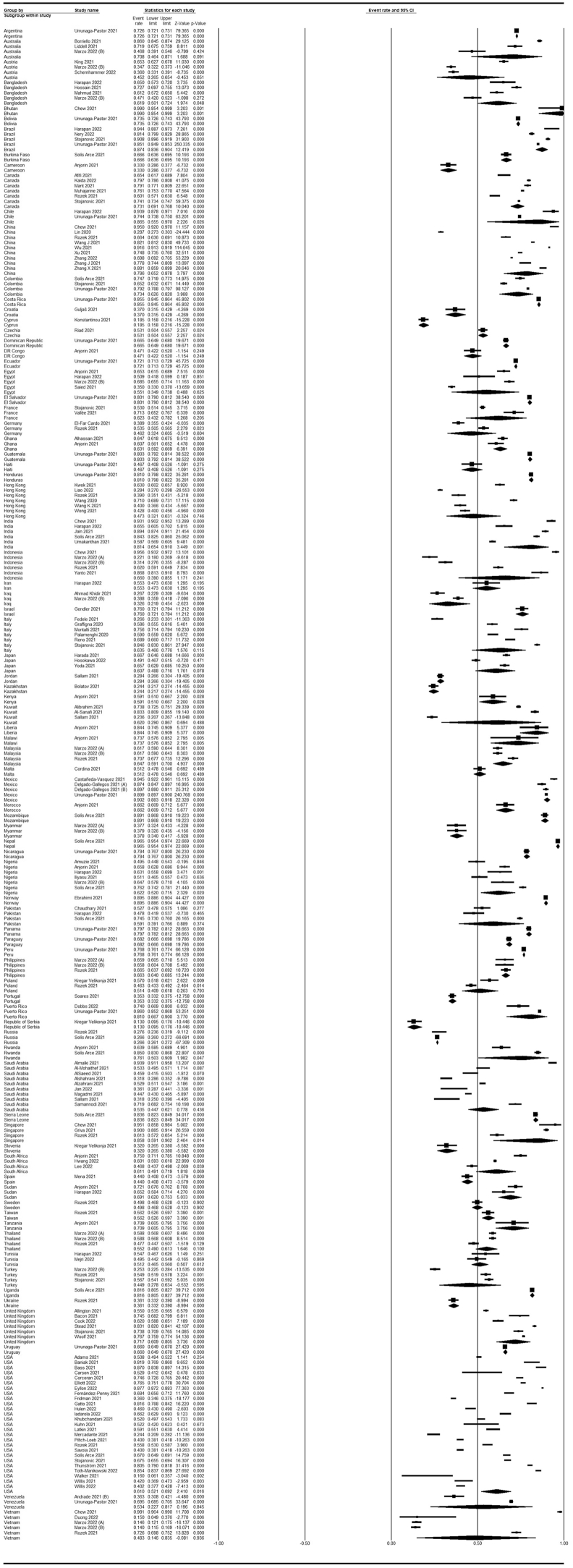
Forest plot displaying acceptance rate of included studies categorized by countries.

**Fig. 14:**
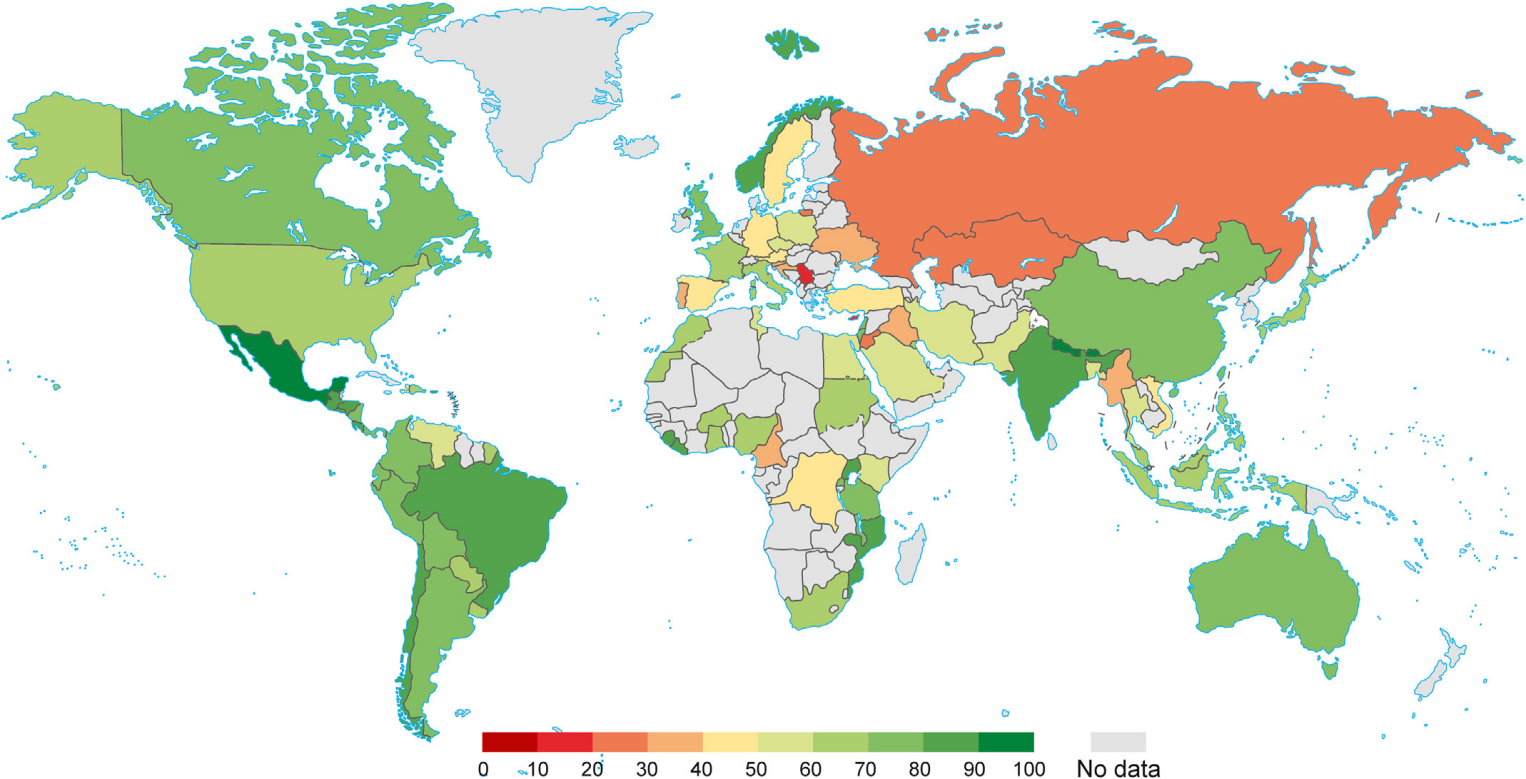
COVID-19 vaccine acceptance rate worldwide.

A meta-analysis of 114 studies encompassing 849,911 participants showed an overall acceptance rate of 63.1% (59.3–66.7%; Fig. 12). Moreover, Fig. 13 shows the acceptance rate by country, and Fig. 14 shows a map of the acceptance rate worldwide.

All analyses were performed using a random-effects model due to significant differences observed in the design, setting, and population of the included studies. In addition, the (I^2^) for all analyses was greater than 50%, confirming heterogeneity among the included studies. Furthermore, applying Duval and Tweedie's trim-and-fill method altered the results for age (*OR* = 0.90; 95% *CI=* 0.73–1.10) and healthcare workers (*OR* = 0.95; 95% *CI=* 0.82–1.11). Funnel plots and other publication bias tests are shown in the Supplementary Material (Figs. S1–S11 and Table S1).

## 4 Discussion

Vaccine uptake rate plays a significant role in achieving herd immunity against COVID-19. The basic reproductive number of an infectious disease is used to calculate the level of population immunity required to limit the spread [141]. According to the most recent COVID-19 estimates, a population of 60–75% immune individuals is necessary to prevent the virus from spreading further and infecting the community [[142], [143], [144]].

Three factors influence vaccination acceptance: complacency, confidence, and convenience [145]. Complacency refers to the belief that the risk of developing a specific disease is low, making immunization unnecessary and avoidable [146,147]. The level of faith and trust in the safety and effectiveness of vaccination is referred to as confidence. The comfort afforded by the population in terms of vaccine accessibility, price, and availability is referred to as convenience [146].

The findings showed that reasons for hesitation are more frequently associated with distrust of medical authorities and vaccine safety. Other factors related to the perception of health risks, such as fear of consequences and lack of information, are also important in vaccine hesitancy. Hence, future vaccination campaigns should emphasize the importance of the individual and include activities aimed at increasing their health knowledge. These actions should be performed at all levels of the healthcare system to increase awareness and trust.

The rapid development of effective and safe COVID-19 vaccines was unprecedented [[148], [149], [150], [151]]. Nonetheless, COVID-19 vaccine apprehension could be a stumbling block in worldwide attempts to contain the pandemic's harmful health and socioeconomic consequences [[152], [153], [154]]. The cost, effectiveness, and duration of protection provided by vaccines appear to be important factors in achieving this goal [150,155,156]; however, vaccine reluctance could be a major obstacle in successfully controlling the COVID-19 outbreak [35].

Consequently, estimates of vaccine acceptance rates can help plan actions and intervention measures to raise public awareness and reassure people about the safety and benefits of vaccines, which can help control the virus’ spread and mitigate the negative effects of this unprecedented pandemic [157,158]. Evaluation of the attitudes toward and acceptance rates of COVID-19 vaccines can aid in launching much-needed communication initiatives to boost public trust in health authorities. Using the results of several COVID-19 vaccine surveys conducted worldwide, this systematic review aimed to analyze the prevalence and factors influencing COVID-19 vaccine acceptance, intention, and hesitancy.

Pogue et al. found that income did not affect vaccination attitudes. Participants with a low educational level also had a lower acceptance rate [153]. These findings are partially in accordance with those of Danis et al. , who found that economic hardship was a driver of vaccination reluctance; however, no link was found between financial hardship and vaccine rejection [159]. By contrast, parental education was a valid predictor of vaccination refusal in both mothers and fathers, whereas reluctance appeared to be unaffected by parental education. In addition, Black and African populations had lower acceptance rates in our study—a finding consistent with another study that found a higher level of skepticism and anxiety regarding the flu vaccine among African Americans [159]. In contrast, our analysis indicates that income does have an impact on vaccination attitudes, with the high-income population showing lower COVID-19 vaccine hesitancy than the low-income population.

Vaccine acceptability among healthcare personnel yielded mixed findings. In general, healthcare workers had higher acceptance; however, Dror et al. found no significant differences in vaccine acceptance between healthcare and non-healthcare personnel in their study, and Barello et al. found no significant differences between healthcare and non-healthcare students [160,161]. Our analysis found a statistically significant difference in COVID-19 vaccine hesitancy between healthcare and non-healthcare workers, with healthcare workers showing less hesitancy than non-healthcare workers. The impact of political ideology on vaccination acceptance or rejection has been one of the most intriguing aspects in some studies; for instance, Kennedy et al. conducted a study focusing on populist parties, finding that—at least in the Western European setting—populist party support might be used as a proxy for vaccination reluctance [162].

The constant advancement in technology suggests that the future of healthcare will be integrated with technology. Therefore, to combat vaccine hesitancy, it is critical to promote population-based communication and information strategies. These strategies include forging multidisciplinary alliances among healthcare providers, providing medical and scientific communications on vaccination, sharing recent data and shreds of evidence on virtual media or brochures, and increasing opportunities for dialogue and counseling regarding vaccination [10,163].

Finally, when the effects of gender and age on COVID-19 vaccination apprehension were examined, it was found that men were more likely to be immunized against COVID-19. This may be because of their stronger perception of COVID-19 hazards and weaker beliefs in disease-related conspiracies [[164], [165], [166]]. As sampling bias—particularly in gender distribution—might alter the reported rates, these variables should be addressed for the appropriate interpretation of COVID-19 acceptance rates. According to our review, men have a higher acceptance rate than women. This finding is consistent with previous research, which indicated that a substantial percentage of women are concerned about vaccination safety and have little faith in the quality and impartiality of the information supplied by healthcare experts [167]. Furthermore, according to our analysis, age was not associated with vaccine acceptance. The results of our study are inconsistent with those of prior research, which demonstrated that the COVID-19 vaccine acceptance rate increased with age [168]. Similar to our results, another study conducted by Salibi et al. among Syrian refugees showed that vaccine rejection did not differ with age [169]. We also analyzed two other sociodemographic factors in our review: marital status and place of residence. According to our results, married people had a lower level of vaccine hesitancy than single people. Moreover, rural people showed a higher rate of vaccine hesitancy than those living in urban areas.

We also analyzed the distribution of vaccine hesitancy and acceptance rates among different countries. The COVID-19 vaccination uptake rates in the Middle East were among the lowest worldwide, with Kuwait (23.6%), Jordan (28.4%), and Saudi Arabia (64.7%) having the lowest acceptance rates [164,170]. Such low rates could be attributed to the region's broad adoption of conspiracy views as well as its subsequent anti-vaccination attitude [165,166,171,172]. Nevertheless, a few nations in the area (such as Israel and the United Arab Emirates) were able to attain vaccination coverage rates that were among the highest in the world, which was ascribed to major efforts to increase vaccine trust [173,174]. The vaccine acceptance rates were relatively high in Latin America, with results from Brazil and Ecuador reporting acceptance rates >70% [175,176]. This was also observed in a survey in Mexico with a vaccine acceptance rate of 76.3% [175]. Urrunaga-Pastor et al. attributed this to the fact that the region was one of the most affected by the pandemic internationally, with high mortality rates per person, which might have contributed to lower levels of complacency [177,178]. High rates of COVID-19 vaccination hesitancy have been reported in Western and Central Europe, with some European countries (Ireland, Italy, Norway, and the United Kingdom), Canada, and the United States having a better outlook. According to a recent study on vaccine hesitancy in the United States for COVID-19, geographic disparity in vaccine hesitancy is closely linked to socioeconomic variables such as race and income. The authors argue that policymakers, community groups, and religious leaders play important roles in building public trust and reducing vaccine-related hesitancy [179].

Furthermore, data from African nations revealed significant rates of COVID-19 vaccination apprehension, particularly in Cameroon (15%) and Senegal (21%); this was mostly because of a lack of confidence in foreign institutions and pharmaceutical corporations [180]. The findings also highlight the need for further African research on COVID-19 vaccination reluctance as information shortages still exist in various African nations.

This study has some limitations. First, the studies included in this analysis varied in population, making comparison of the results challenging. Second, most studies have relied on self-reported surveys, which increased the risk of response bias.

Third, it is crucial to consider that people's views on vaccines may change as real-world data become available. The studies included in our review captured public opinions during the peak of the COVID-19 pandemic, a time when information related to vaccines was still emerging and often scarce.

Further research is required to confirm this hypothesis. Longitudinal studies that follow individuals over time could be valuable for understanding how attitudes evolve with new developments. Directly conducting interviews and focus groups with individuals can provide insights into beliefs and concerns that surveys may overlook.

## 5 Conclusion

Being men, living in an urban region, married, educated, having a history of influenza vaccination, having a higher income level, and having a history of comorbidities were associated with higher COVID-19 vaccine acceptance. In contrast, older age, history of prior COVID-19 infection, and being a healthcare worker did not significantly change the COVID-19 vaccine acceptance rate.

## Author contributions

M.B., F.F., M.R., and M.A. participated in the search strategy and research design of systematic review. M.B and F.F. did the articles screening of titles and abstracts. F.G., R.R., H.B., and F.S. did the article screening of full texts and data extraction. Any disagreements were resolved by consulting with a third reviewer (AL, NS, and MA). A.L. did the meta-analysis. A.L., N.S., F.G., R.R., H.B., and F.S. drafted the manuscript. A.L., N.S., and M.R. critically revised the manuscript for important intellectual content and approved the final version to be published. M.A. supervised the study and revised the manuscript for important intellectual content.

All authors have read and approved the manuscript.

## Data statement

All data are available by the corresponding author upon reasonable request.

## Funding

This research did not receive any specific grant from funding agencies in the public, commercial, or not-for-profit sectors.

## Declaration of competing interest

The authors declare that they have no known competing financial interests or personal relationships that could have appeared to influence the work reported in this paper.
