# Supplementary material for: COVID-19 vaccine hesitancy worldwide and its associated factors: a systematic review and meta-analysis
Source: Sci One Health. 2023 Nov 14;2:100048. doi: 10.1016/j.soh.2023.100048 (PMC11262288; doi:10.1016/j.soh.2023.100048)
Supplement: Multimedia component 1 [file mmc1.docx]

| Analyzed Factors | Egger's test (p value) | Studies trimmed | Before trim and fill | After trim and fill |
| --- | --- | --- | --- | --- |
|  |  |  | OR of hesitancy (95% CI) | OR of hesitancy (95% CI) |
| Male | 0.001 | 1 | 0.75 (0.70–0.79) | 0.74 (0.70–0.79) |
| Older ages | 0.232 | 7 | 0.76 (0.62–0.92) | **0.90 (0.73–1.10)** |
| Living in urban areas | 0.720 | 3 | 0.74 (0.64–0.85) | 0.65 (0.56–0.75) |
| Married | 0.212 | 0 | 0.80 (0.69–0.94) | 0.80 (0.69–0.94) |
| Educated | 0.832 | 1 | 0.81 (0.72–0.91) | 0.81 (0.72–0.90) |
| Higher income | 0.690 | 5 | 0.57 (0.46–0.69) | 0.48 (0.39–0.59) |
| Positive COVID-19 history | 0.998 | 0 | 1.18 (0.90–1.55) | 1.18 (0.90–1.55) |
| History of flu vaccination | 0.396 | 2 | 0.41 (0.34–0.51) | 0.38 (0.30–0.47) |
| Healthcare workers | 0.222 | 3 | 0.86 (0.75–0.99) | **0.95 (0.82–1.11)** |
| Having Comorbidities | 0.312 | 3 | 0.84 (0.77–0.91) | 0.82 (0.75–0.89) |

**Table S1.** Trim and fill, and Egger’s test for publication bias results

OR, Odds Ratio, COVID-19; 95% CI, 95% confidence intervals; Coronavirus Disease 2019

**Fig. S1**: Funnel plot for male gender

**Fig. S2**: Funnel plot for older ages

**Fig. S3**: Funnel plot for living in urban areas

**Fig. S4**: Funnel plot for married individuals

**Fig. S5**: Funnel plot for educated individuals

**Fig. S6**: Funnel plot for high-income individuals

**Fig. S7**: Funnel plot for positive history of COVID-19

**Fig. S8**: Funnel plot for history of flu vaccination

**Fig. S9**: Funnel plot for healthcare workers

**Fig. S10**: Funnel plot for individuals with comorbidities

**Fig. S11.** Funnel plot for acceptance rate
